# Whether Prolyl Hydroxylase Blocker—Roxadustat—In the Treatment of Anemia in Patients with Chronic Kidney Disease Is the Future?

**DOI:** 10.3390/ijerph18041612

**Published:** 2021-02-08

**Authors:** Władysław Grzeszczak, Dariusz Szczyra, Mirosław Śnit

**Affiliations:** Department of Internal Medicine, Diabetology and Nephrology, Faculty of Medical Sciences in Zabrze, Medical University of Silesia, Poniatowskiegostr., 40-055 Katowice, Poland; dszczyra@sum.edu.pl (D.S.); msnit@sum.edu.pl (M.Ś.)

**Keywords:** chronic kidney disease, anemia, roxadustat

## Abstract

In patients with chronic kidney disease (CKD), anemia develops gradually, which is primarily due to an inadequate synthesis of erythropoietin by the kidneys, as well as to iron disorders in the body, blood loss, shortened erythrocyte survival and inflammation. The currently accepted treatment employs iron, vitamin B12, folic acid supplementation and the use of erythropoiesis stimulants, which are administered only parenterally. Research is currently underway on the new erythropoiesis drugs that can be orally administered, i.e., hypoxia-inducible factor-propyl hydroxylase inhibitor (HIF-PHI) inhibitors which temporarily block propyl hydroxylase [PHD] catalysis and promote a transient increase in the expression of genes regulated by HIF, including kidney and liver erythropoietin [EPO]. Roxadustat is the first oral drug in this class and a potent HIF-PHD inhibitor, exerted to treat anemia in patients with CKD. In phase 1, 2 and 3 studies with CKD-affected patients, roxadustat was more effective to stimulate erythropoiesis for anemia correction than previously used drugs. Roxadustat can be orally given, unlike other erythropoiesis drugs with parenteral administration only, which grants roxadustat a considerable advantage. Our paper presents the results of studies with roxadustat applied for the treatment of anemia in CKD patients with or without dialysis. We are currently not yet able to know the exact role of roxadustat in the treatment of anemia in patients with CKD, but time will tell. It is possible that roxadustat has benefits an iron metabolism and cardiovascular risk.

## 1. Introduction

Anemia is a disorder in which hemoglobin, hematocrit (Ht) and erythrocyte levels in the blood are reduced by >2 standard deviations vs. their normal values. Smaller erythrocyte numbers and reduced hemoglobin concentrations lead to decreased amounts of oxygen, transported to tissues and organs throughout the body, which translates directly into their compromised functioning [1].

The link between uremia and anemia was first described by Richard Bright almost 200 years ago [2]. Anemia is common in patients with chronic kidney disease (CKD). The incidence of anemia increases as the disease progresses. In patients with chronic kidney disease (CKD), anemia develops gradually, primarily due to inadequate renal synthesis of erythropoietin, disturbances in iron balance in the body (which may be in part due to increased hepcidin levels), blood loss, decreased red blood cell survival and inflammation [3,4].

It is thus obvious that the treatment of CKD-induced anemia is a clinically significant issue. The current treatment protocols use iron, vitamin B12, folic acid supplementation and only parenterally administered erythropoiesis stimulants.

Research is currently underway on new erythropoiesis drugs that could be orally given. When oxygen deficiency occurs, the oxygen homeostasis-regulating factors are activated in the cells. The main factors, responsible for the cellular responses to changes in oxygen deficiency, include hypoxia-inducible factors (HIFs), with two isoforms, HIF1α and HIF2α, prolyl hydroxylase (PHD) with three isoforms: PHD1, PHD2 and PHD3, aspartic hydroxylase (asparaginyl hydroxylase) and the factor inhibiting hypoxia-inducible factor 1α (HF) [4,5,6].

Hypoxia-induced factor (HIF) is a heterodimeric transcription factor responsible for stimulating the secretion of erythropoietin [EPO] and other oxygen-deficient genes. HIF prolyl hydroxylase (HIF-PHD) enzymes significantly affect the stability of the HIF-α subunit of the HIF transcription factor by promoting post-translational hydroxylation of HIF in an oxygen-dependent manner. This contributes to the balance between oxygen availability and HIF activity.

**Figure 1:**
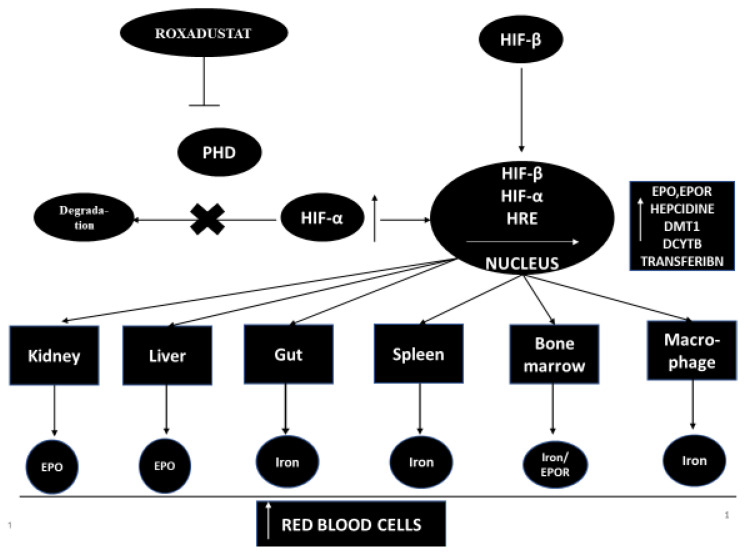
The mechanism of action of the HIF-PHD inhibitor. DCYTB—duodenal cytochrome b reductase 1, DMT1—divalent metal transporter-1, EPO—erythropoietin, EPOR—erythropoietin receptor, HIF—hypoxia-inducible factor, HRE—hypoxia response element, PHD—propyl-4-hydroxylase domain protein.

Hypoxia-inducible factor-propyl hydroxylase inhibitor (HIF-PHI)temporarily inhibits PHD catalysis and contributes to a transient increase in HIF expression, and effects the function of many genes, including kidney and liver EPO (or EPO/EPO receptor, proteins promoting iron absorption, iron transport and heme synthesis) [7,8]. However, the increase in plasma EPO concentration in patients with end-stage renal disease treated with the full therapeutic success of HIF-PHI is significantly lower than in patients treated with recombinant human erythropoietin [rhEPO] injections [9]. HIF-PHI has a beneficial effect on iron homeostasis as it reduces the level of hepcidins and ferritin and increases the total iron binding capacity (TIBC) in patients with end-stage renal disease [9]. HIF-PHI inhibitors are drugs that can become an alternative therapy to conventional erythropoiesis stimulating agents [ESA]. It has been shown that, in addition to stimulating erythropoiesis, HIF-PHI inhibitors have pleiotropic effects that affect cholesterol levels and blood pressure [9,10,11]. The mechanism of action of this medicine is shown in Figure 1.

Roxadustat (Ai RuiZhuo^®^ in China) is the first oral drug in its class and a potent HIF-PHD inhibitor used to treat anemia in CKD patients not on dialysis and on dialysis, as well as in myelodysplastic syndromes.

Roxadustat has received approval in China to improve the severity of anemia in patients with CKD.

The FibroGen Company has submitted an application to the Food and Drug Administration [FDA] for permission to use roxadustat in patients with chronic renal failure accompanied by anemia [12]. The application is under consideration.

### 1.1. A Little Information about Roxadustat

Roxadustat was developed almost a decade ago [13]. Roxadustat (FG-4592) is a new oral drug that stimulates the synthesis of endogenous erythropoietin and also affects and regulates the body’s iron metabolism [14]. Roxadustat temporarily binds and significantly reduces the activity of HIF-PHD enzymes. This leads to a decrease in the degradation of HIF-α and to an increase in the transcriptional activity of HIF [11]. Increased HIF activity stimulates other genes that take an active part in erythropoiesis, such as the EPO gene, the EPO receptor gene, protein genes promoting iron absorption, iron transport and heme synthesis [11,14]. Roxadustat increases the level of hemoglobin in a dose-dependent manner and significantly reduces hepcidin levels [13]. Roxadustat significantly affects iron metabolism in patients with CKD, increasing serum transferrin levels and improving the intestinal absorption of iron in patients with CKD and anemia [9,13,15,16].

### 1.2. Pharmacokinetics of Roxadustat

Roxadustat clinical trials started in November 2005 [14]. The pharmacokinetics of roxadustat were studied in healthy Caucasian and non-Caucasian subjects. Those studies showed that, after the administration of roxadustat, the mean blood hemoglobin concentration was similar in Caucasians and other races. There was no accumulation of the drug after its repeated, three-times-a-week administration [17]. Neither food intake [18] nor moderate hepatic impairment affected the pharmacokinetics of the drug (NCT02805374) [12]. In another study (NCT 02252731), roxadustat had no significant effect on warfarin pharmacokinetics when both Lin et al. [19] drugs were simultaneously given.

## 2. Roxadustat—Phase 1 Studies

The first study to evaluate roxadustat (FG-4592) as an oral instructional hypoxia prolyl hydroxylase inhibitor that stimulates erythropoiesis was conducted in patients with end-stage therapy failure and anemia and treated with hemodialysis. Seventeen hemodialysis patients who received erythropoietin alfa during treatment and who maintained hemoglobin levels ≥10 g/dg/dL were enrolled in the study. Erythropoietin alfa was discontinued 3 days before the start of the study. After 3 days, the subjects were entered into a double-blind, placebo-controlled trial. The subjects were randomized into two cohorts, 3:1 (roxadustat:placebo). Patients received roxadustat (1 or 2 mg/kg) or placebo one hour after hemodialysis on day one and two hours before dialysis on day eight. Peak plasma concentrations and area under the roxadustat curve were dependent on dose and time post-dose. The half-life of roxadustat ranged from 14.7 to 19.4 h. Roxadustat was shown to be very highly bound to plasma proteins (99%). Dialysis alone had little effect on the final drug clearance (only 4.56% and 3.04% roxadustat by dose). Administration of roxadustat resulted in increased levels of endogenous erythropoietin. Peak erythropoietin levels following roxadustat administration were found between 7 and 14 h after dosing. Erythropoietin levels returned to baseline values 48 h after administration of roxadustat. The obtained maximum concentration of erythropoietin was dose-dependent and was 96 mIU/mL after administration of roxadustat at 1 mg/kg and 268 mIU/mL at 2 mg/kg. Similar concentrations of erythropoietin are found in patients after heavy blood loss or in people staying at high altitudes above the ground. No serious adverse effects were found [20].

## 3. Roxadustat—Phase 2 Studies

Phase 2 clinical studies of Roxadustat were started in December 2006. Roxadustat was applied in a few randomized, open phase 2 studies in patients with anemia and terminal renal failure not treated by hemodialysis [9,13,15] and treated by hemodialysis [8,10]. The results of roxadustat administration to maintain Hb levels were at least as effective as those after alpha erythropoietin.

### 3.1. Roxadustat in Patients with Terminal Renal Failure Not Treated by Dialysis

A randomized, double-blind, phase 2 study, involving not dialysis-dependent patients with anemia and CKD (NCT01599507) [21], showed that roxadustat, when used three times a week, increased Hb levels during the 8-week study period, with a significant difference between the roxadustat groups, receiving either a low dose (1.1–1.75 mg/kg) or a high dose (1.5–2.25 mg/kg), and a placebo group (the mean change +1.82 and +2.59 vs. 0.65 g/dL; both *p* < 0.01) [12] (see Table 1) [9].

A randomized, single-person, placebo-controlled, phase 2a study (NCT00761657; *n* = 116) in patients with anemia and terminal renal failure, not treated by hemodialysis, showed that roxadustat, used three times a week, increased Hb levels by dose (0.8–2.2 mg/dg/dL) during the 6-week study period (4 weeks of treatment), with a significant difference between the roxadustat groups of 1.5 and 2.0 mg/kg, compared to a placebo group (the mean change +1.2 and +1.8 vs. −0.1 g/dL; both *p* < 0.01; [10]) (Table 1) [13].

In a randomized, open, multicenter, phase 2b clinical trial (NCT01244763), roxadustat, administered in different initial doses and at different frequencies for 16 or 24 weeks, achieved anemia correction and decreased serum hepcidin levels in 143 patients with evaluated anemia (baseline Hb ≤ 10.5 g/dL) and CKD, not treated by hemodialysis [10]. After 16 weeks of roxadustat treatment, 92% of the patients demonstrated a Hb response, defined as the percentage of patients with increased Hb ≥ 1.0 g/dg/dL, compared to baseline, and Hb ≥ 11.0 g/dg/dL by the end of week 16 (main endpoint) (see Table 1). The treatment with roxadustat significantly reduced hepcidin levels by 17% (*p* = 0.004), increased Hb levels by an average of 1.83 g/dg/dL (*p* < 0.001) and maintained the Hb reticulocyte content during the first 16 weeks of treatment. The average total cholesterol was also significantly reduced (*p* < 0.001) by 26 mg/dL after 8 weeks of treatment with roxadustat, regardless of the use of statins or other lipid-lowering agents [11].

In a phase 2, double-blind study (NCT 01964196), patients with renal failure not undergoing dialysis {NDD-CKD] were randomized to oral placebo or roxadustat administration regimes (50, 70, or 100 mg), three times weekly (TIW) for 6 weeks. Out of 107 randomized patients, 83 completed the study. See Table 1 for the obtained results. No deaths or major adverse cardiac events were recorded after roxadustat. Tadao Akizawa et al. [22] stated that roxadustat, while being well-tolerated, had been effective in correcting Hb levels in Japanese anemic NDD-CKD patients during the 6-week therapy.

### 3.2. Roxadustat in Patients with Terminal Renal Failure Treated by Dialysis

Bessarab et al. [23] were the authors of a randomized, open-label study of the course of hemoglobin (Hb) leveling after roxadustat administration in patients with anemia (Hb <10.0 g/dL) treated with hemodialysis (HD) or peritoneal dialysis (PD). The study included sixty patients treated with dialysis, who received roxadustat for 12 weeks. The baseline Hb concentration in patients was 8.36 g/dg/dL. In subjects, roxadustat was used in gradually increasing doses. After seven weeks, the Hb concentration increased by 2.0 g/dL in the subjects. After 12 weeks of treatment, the mean maximum change in Hb from baseline in patients was 3.16 g/dL (*n* = 55). In the groups of patients additionally treated with iron (orally or intravenously), the mean maximum change in Hb level from the baseline value was similar and was greater than in the group of patients not treated with iron. Increase in Hb of ≥1.0 g/dL (from baseline) was observed in 96% of patients. Roxadustat, being well-tolerated, corrected anemia in HD and PD patients.

The study (NCT01147666) was a two-part study and included hemodialysis-treated anemia and terminal renal failure patients who had previously received erythropoietin and intravenous iron for 4 weeks prior to randomization, showing an average Hb level of 9–13.5 g/dL for 8 weeks [10]. In the first part of the study (over 6 weeks), 54 patients received different doses of roxadustat three times a week (1–2 mg/kg fixed doses) compared with continuous intravenous administration of erythropoietin alfa. The increase in hemoglobin was significantly greater in patients receiving roxadustat 1.5–2 mg/kg (total) than in patients receiving alpha erythropoietin (79% vs. 33%) (the primary endpoint was response to treatment Hb (ΔHb) change by −0.5 g/dL). Moreover, the subjects showed a significant reduction in the level of hepcidin (*p* < 0.05) in patients treated with roxadustat (2 mg/kg) compared to patients treated with erythropoietin alfa [9]. In the second part of this study, 90 patients were treated for 19 weeks with roxadustat, divided into six cohorts. Sixty-seven subjects received roxadustat three times a week at various starting doses and according to different rules (1.0–2.0 mg/kg, or depending on body weight), while the remaining 23 patients continued treatment with erythropoietin alfa [16]. In an evaluation of the efficacy of the treatment, 51% (31/61) of patients receiving roxadustat achieved an average Hb level of ≥11 g/dg/dL in the last 4 weeks of the 19-week treatment period. Of those treated with alpha-erythropoietin, only 36% (8/22) achieved a mean Hb level of ≥11 g/dL in the last 4 weeks of the 19-week treatment period (primary endpoint) [10].

### 3.3. Summary

The presented data from six published phase 2 clinical trials indicate that roxadustat was able, on the one hand, to increase the level of Hb, and, on the other hand, to maintain the level of Hb at an elevated level, both in patients not on dialysis and in patients undergoing dialysis with CKD [8,10,13,15,21]. More than 40% of the respondents had diabetes. As has been shown, roxadustat has a positive effect on iron metabolism (it was manifested by a significant reduction in the level of hepcidin in the serum). Recovery of the severity of anemia with roxadustat was independent of changes in the degree of pre-existing inflammation. In addition, roxadustat (including other HIF-PHI) has been shown to cause other effects as well. These include lowering the level of low-density lipoprotein or serum cholesterol and lowering blood pressure in clinical or preclinical trials in patients with CKD.

## 4. Roxadustat—Phase 3 Studies

Phase 3 clinical studies of roxadustat were started in 2014. There are currently more than 15 phase 3 clinical trials underway with a target enrollment of about 10,000 patients worldwide, studying the safety, efficacy, and long-term effects of roxadustat in CKD patients, including non-dialysis-dependent [23,24,25,26], hemodialysis-dependent [22,25,27,28,29] and peritoneal dialysis-dependent subjects [25,30]. See below for a few presented examples.

### 4.1. Roxadustat in Patients with Terminal Renal Failure, Not Treated by Dialysis

From December 2015 up to September 2016, Chen et al. [16], in China, conducted the third phase of a randomized, double-blind trial with roxadustat (NCT02652819). The study included 154 patients (aged 18–75) with CKD, who had not started renal replacement therapy. Eventually, the study was launched with 151 patients on board.

The patients included in the study with CKD with a baseline hemoglobin concentration between 7 and 10 g/dL had not received any agents stimulating erythropoiesis for at least 5 weeks before [19].

One hundred and thirty-one patients (87 receiving roxadustat and 44 receiving placebo) completed the first stage of the study.

The study was divided into 2 stages. Initially, roxadustat/placebo was administered 3 times a week for 8 weeks (hemoglobin concentration was tested in both groups between the 7th week and the 9th week), and then all the willing patients received roxadustat for a further 18 weeks.

#### 4.1.1. The First Stage of the Study

The main purpose of the first stage was to examine the change in hemoglobin concentrations between weeks 7 and 9 of the study.

After 9 weeks, patients with CKD not undergoing dialysis treated by roxadustat observed increased hemoglobin level by 1.9 ± 1.2 g/dg/dL. In the placebo group, this increase was only 0.4 ± 0.8 g/dL.

An increase in Hb concentration by more than 1 g/dg/dL after 9 weeks of the study was observed in 85/101 patients receiving roxadustat (84%), while no patient on placebo achieved such an increase in hemoglobin level. In 68 patients with CKD treated with roxadustat (68%) and in 3 patients not treated with roxadustat (6%), the mean hemoglobin level was found to be at least 10 g/dg/dL between the seventh and ninth weeks of treatment. Hemoglobin concentration of 10 g/dg/dL and more in the patients with hemoglobin increase of at least 1 g/dg/dL (with the baseline hemoglobin level above 8 g/dg/dL), and in patients with baseline Hb levels below 8 g/dg/dL, an increase in hemoglobin levels by at least 2 g/dg/dL was reported in 76 patients from the roxadustat group (75%), while none of the patients receiving placebo achieved that result. An emergency therapy was used in 3 patients receiving roxadustat (3%) and 6 in the placebo group (12%) [16].

#### 4.1.2. The Second Stage of the Study

After completing the double-blind, randomized study, 131 patients (87 previously taking roxadustat and 44 receiving placebo) continued the study. The assumption was that each of them had received roxadustat for 18 weeks. The second part of the study was completed by 98 patients. The patients, who received roxadustat, had previously maintained stable hemoglobin levels and, at the end of week 26 of the study, 71/85 (84%) had the hemoglobin level higher than 11.0 g/dg/dL. In the placebo group, after switching to roxadustat, hemoglobin levels above 11.0 g/dg/dL were observed in 31/43 patients (72%) [16]. Total and low density cholesterol-LDL levels also decreased, especially in the group that had previously received a placebo. At the end of the study, all the subjects had similar results for total and LDL cholesterol [16]. Two deaths were recorded during the study.

The randomized, double-blind, placebo-controlled phase III study (NCT01750190; *n* = 922) called ANDES enrolled patients with chronic kidney disease in stages 3, 4 or 5 CKD, not under dialysis [12]. The subjects were divided into two groups: roxadustat-treated and placebo-treated. The authors showed that roxadustat was effective in the treatment of anemia (as assessed by the primary endpoints in the US and EU). In the presented study, the mean duration of treatment was 1.7 years, and the mean baseline Hb level was 9.1 g/dL in both groups, i.e., roxadustat or placebo. For the primary endpoint in the US study, the mean increase in Hb from baseline to that observed after 28–52 weeks was significantly greater in the roxadustat group than in the placebo group (2.0 vs. 0.16 g/dL respectively; *p* < 0.0001). For the primary endpoint of the EU study, the goal was to achieve an Hb concentration of ≥11 g/dL and an increase in Hb of ≥1 g/dL. A significantly higher percentage of patients in the roxadustat group, as opposed to the placebo group, achieved the expected Hb value (86 vs. 7%; *p* = 0.0007). In a pre-defined secondary analysis, roxadustat significantly reduced the risk of salvage treatment by 81% compared to placebo (HR 0.19; *p* < 0.0001), where lifetime therapy was defined as erythropoiesis stimulating agents [ESA] or intravenous iron during the first 52 weeks of treatment. In patients treated with roksadustat, the risk of requiring the first blood transfusion in the first 52 weeks decreased by as much as 74% (HR 0.26; *p* < 0.0001) [12].

Patients with CKD, un-dialyzed and anemic, were enrolled in the randomized, double-blind, placebo-controlled phase 3 ALPS [Astellas Pharma Study] study (NCT01887600). The authors showed that in patients treated with roxadustat, a significantly greater increase in Hb levels was observed both in the first 24 weeks of treatment and in weeks 28–52 (interrelated endpoints) [17].

In a phase III randomized, double-blind, placebo-controlled study (NCT02652819; *n* = 152), treatment was conducted in Chinese anemic and non-dialyzed Chinese CKD patients. The baseline Hb concentration was 7–10 g/dL. In patients treated with roxadustat, a significant increase in Hb levels was demonstrated [31]. After 8 weeks of treatment, three times a week, the mean change in Hb from baseline was significantly greater in the roxadustat group than in the placebo group (1.9 vs. 0.39 g/dg/dL, respectively; *p* < 0.0001).

In the HIMALAYAS randomized, open, active-controlled, phase III comparator study (NCT02052310; *n* = 1043), roxadustat was not worse than alpha erythropoietin in the treatment of anemia in patients who had recently started treatment with hemodialysis, due to terminal renal failure [12].

The treatment lasted on average 1.8 years, and the mean baseline Hb concentration in the patients was about 8.4 g/dg/dL. The examined patients with CKD were divided into the group treated with roxadustat and the group treated with erythropoietin alfa. For the primary endpoint in the US study, the mean increase in Hb after 28–52 weeks of treatment was shown to be 2.57 g/dL for roxadustat-treated patients and 2.36 g/dL for erythropoietin alfa-treated patients. It has been shown that patients treated with roxadustat compared to patients treated with alpha erythropoietin had higher Hb levels (*p* = 0.0005) [12].

Akizawa et al. [32] presented data from a multicenter, randomized, open-label phase III study. The study included 100 patients with CKD, not undergoing dialysis, previously treated with erythropoiesis-stimulating drugs. Subjects were randomized to roxadustat (starting dose 50 or 70 mg, 3 times per week) adjusted to maintain hemoglobin (Hb) levels between 10.0 and 12.0 g/dL for ≤24 weeks. After 18–24 weeks of treatment, there was an increase in concentration of 1.34 g/dg/dL. The authors concluded that roxadustat is an effective drug in the correction of anemia in patients with CKD not previously treated with ESA, with iron deficiency.

### 4.2. Roxadustat in Patients with Terminal Renal Failure, Treated by Hemodialysis or Peritoneal Dialysis

Chen et al. [18] (NCT02652819) conducted a parallel study in patients with CKD treated with renal replacement therapy (hemodialysis or peritoneal dialysis), comparing the effectiveness of roxadustat and erythropoietin alfa: 305 patients were enrolled into the study, all of them with end-stage renal insufficiency and on hemodialysis or peritoneal dialysis treatment for at least 16 weeks, aged between 18 and 75 years. The patients were randomized at the 2:1 ratio for oral roxadustat (204 patients) and alpha parenteral erythropoietin (101 patients). Roxadustat 100 mg was used in patients weighing 45–60 kg, and roxadustat in a dose of 120 mg was used in patients weighing more than 60 kg. The patients were also divided into those taking alpha erythropoietin at a dose of <8000 international units [IU] per week, those taking ≥8000 IU per week, as well as those treated with hemodialysis and treated with peritoneal dialysis. Oral iron preparations were authorized. The use of intravenous iron preparations, red blood-cell-concentrate transfusions and an additional stimulation of erythropoiesis (or a combination of those methods) was allowed only as a life-rescue therapy in the patients with hemoglobin drop below 8 g/dg/dL or less than 9 g/dg/dL if the decrease was more than 1 g/dg/dL from the assumed starting point of 10.4 g/dg/dL. The study lasted 26 weeks.

The primary goal was to assess changes in hemoglobin concentrations in the 23rd week and in the 27th week after the start of the study.

At the end, 305 patients took part in the study (204 received roxadustat and 101 erythropoietin alpha): 48 patients (42 receiving roxadustat and 6 receiving erythropoietin alpha) discontinued the medication before the study was completed. Eventually, 254 patients (162 from the roxadustat group and 96 from the alpha erythropoietin group) completed the study [18].

Hemoglobin levels were shown to increase by 0.7 ± 1.1 g/dg/dL in the roxadustat group, of which 92.5% (189 subjects) increased by less than 1 g/dg/dL and 0.5 ± 1.0 g/dg/dL in the alpha erythropoietin group, of which 92.5% (92 subjects) increased by less than 1 g/dg/dL. In 87% (178 patients) of the group of CKD patients treated with roxadustat and in 88.5% (88 patients) of the group of CKD patients treated with alpha-erythropoietin, hemoglobin levels exceeded10 g/dL [18].

The change in blood pressure in the roxadustat group was −2.1 and −0.7 mmHg, compared to the alpha erythropoietin group [18].

In another randomized, open-label, phase III study (SIERRAS—NCT02273726; *n* = 741), which included anemic CKD patients, roxadustat turned out to be a less effective drug than alpha-erythropoietin [12]. The mean duration of follow-up was 1.9 years, and the baseline Hb level in both treatment groups was 10.3 g/dg/dL. In a US study, the mean increase in Hb after 28–52 weeks was 0.39 g/dg/dL in the group of CKD patients treated with roxadustat compared to 0.09 g/dg/dL in the group of CKD patients treated with erythropoietin alfa. In an EU study, the mean increase in Hb after 28–36 weeks was 0.54 g/dg/dL in the group of CKD patients treated with roxadustat compared to0.02 g/dg/dL in the group of CKD patients treated with erythropoietin alfa [12].

Chen et al. [31], conducting a phase III study (NCT02652806; *n* = 304) in patients with CKD treated with repeated hemodialysis or peritoneal dialysis, and for the last 6 weeks ESA, showed that roxadustat was as effective as erythropoietin alfa in the treatment of anemia. The starting Hb concentration was 10.4 g/dL. Following treatment with roxadustat or erythropoietin alfa, three times a week for 26 weeks (adjusted to maintain/achieve Hb 10–12 g/dL), the mean change in Hb at 23–27 weeks was less for roxadustat than for erythropoietin alfa (0.8 vs. 0.5 g/dg/dL). Roxadustat also significantly increased the level of transferin, maintained normal serum iron levels and weakened the reduction in transferin saturation (TSAT), compared to alpha erythropoietin (all *p* < 0.01) [31].

In a randomized, double-blind, placebo-controlled, phase III study (NCT02952092; *n* = 303), Japanese anemic CKD patients were treated with repeated hemodialysisand received ESA for ≥12 weeks. Patients were treated with roxadustat (at a dose of 70 or 100 mg) three times a week or with darberythropoietin alfa (at a dose of 10–60 μg) once a week (doses adjusted to maintain Hb levels at 10–12 g/dL). The authors showed that that roxadustat maintained Hb levels and was no less effective than darberythropoietin alfa [33]. The mean Hb levels at weeks 18–24 in roxadustat-treated patients were 11 g/dL. The mean Hb levels ranged from 10 to 12 g/dL. Roxadustat was no less effective than darberythropoietin alfa, also because for the lower 95% confidence interval (CI) the difference between roxadustat and darberythropoietin alfa was greater than the weightlessness criterion of 0.75 g/dg/dL [32].

In an open-label, multicenter, phase III study in Japanese patients with chronic end-stage renal failure treated with peritoneal dialysis (NCT02780726; *n* = 56), roxadustat was used to treat anemia (3–7). The authors’ goal was to achieve and maintain the Hb concentration (10.0 and 10.5 g/dg/dL) and increase Hb > 1 g/dg/dL. After the application of roxadustat, the previously mentioned objectives were achieved. Patients previously untreated with ESAs (*n* = 13) were randomized to roxadustat 50 or 70 mg, while patients previously treated with ESAs (*n* = 43) received roxadustat 70 or 100 mg, depending on the previous dose of ESA. Doses were adjusted to maintain Hb levels at 10–12 g/dL at the end of treatment after 18–24 weeks. Target Hb levels after 18–24 weeks of roxadustat treatment were found in 92.3% of ESA-naïve patients and 74.4% of ESA-previously treated patients. The survival rate of patients who achieved at least one Hb target at weeks 18–24 and treatment-naïve was 92% compared with 87% in ESA-previously treated patients. On the one hand, roxadustat turned out to be a useful drug in maintaining a predetermined target concentration of Hb in patients with CKD per PD, who were previously treated or not treated with ESA, as the studies show. On the other hand, roxadustat was well-tolerated by the respondents [33].

In 2019, during Kidney Week of the American Society of Nephrology (ASN) 2019, Washington, USA, detailed results were presented of the following two clinical trials: the phase III 343 OLYMPUS (NCT02174627 clinical trial; *n* = 2781) and the ROCKIES (NCT02174731 clinical trial; *n* = 3442 [15] The presented results showed that roxadustat had significantly increased hemoglobin levels in patients with untreated anemia and treated by hemodialysis. The OLYMPUS study compared the effectiveness of roxadustat improvement in anemia with placebo. In the OLYMPUS study, roxadustat in the treated patients increased Hb levels by an average of 1.75 g/dL, while in the placebo group, the increase was 0.40 g/dL (*p* < 0.0001) after 28–52 weeks of treatment. In the study group, roxadustat also increased Hb levels in the subgroup of patients with elevated levels of C-reactive protein (hsCRP) (>5 mg/L) compared to placebo (1.73 to 0.62 mg/dL). The safety results of roxadustat were similar to the NDD-CKD patient population. The ROCKIES study compared the effectiveness of anemia improvement in the group of patients treated with roxadustat with the group of patients treated with erythropoietin alfa. In the ROCKIES study, roxadustat-treated groups experienced a significant increase in Hb levels (by 0.77 g/dL) compared to 0.68 g/dL in patients treated with erythropoietin alfa after 28 to 52 weeks of treatment. As in the OLYMPUS study, roxadustat also increased Hb levels to a greater extent in the subgroup of patients with elevated levels of C-reactive protein (hsCRP) (>5 mg) compared to the group of patients treated with erythropoietin alfa (0.80 to 0.59 g/dg/dL). Patients treated with roxadustat were shown to receive less intravenous iron per month (59 mg/month) compared with patients treated with erythropoietin alfa (91 mg/month) from 36 weeks to the end of the [15].

## 5. Additional Assessed Parameters

### 5.1. Hepcidin

In humans, the hepcidin antimicrobial peptide [HAMP] gene codes for the synthesis of a protein called hepcidin. In mammals, hepcidin is a protein necessary for iron to enter the circulation. In inflammatory conditions in the human body, hepcidin levels are very high, and serum iron levels decrease due to the spilling of iron by macrophages and hepatocytes, and due to reduced iron absorption from the gastrointestinal tract. In their study, Chen et al. [16] determined the baseline hepcidin concentration and hepcidin concentration after 9 weeks of treatment for non-dialyzed CKD warts. In the group of patients treated with roxadustat, the initial hepciden concentration was 95.89 ng/mL, while in the placebo group, it was 114.71 ng/mL. After 9 weeks of treatment, hepcidin levels decreased by 56.14 ng/mL in the roxadustat group and by 15.10 ng/mL in the placebo group [16]. Summarizing, Chen et al. found that in their group of patients with CKD not treated with roxadustat, there was a significant decrease in heptacidin levels compared to placebo (mean change 56 vs. −15 ng/mL; *p* < 0.00001) [32]. In the patients with CKD, treated by dialysis, Chen et al. [18] observed serum hepcidin basal concentrations to be 180.7 ± 136.8 ng/mL and changing at week 23 of the study by −30.2 ± 113.3 ng/mL in the roxadustat group, while in the alpha erythropoietin group, serum hepcidin basal concentrations were 148 ± 105.2 ng/mL and changed by −2.3 ± 130.7 ng/mL [18].

Serum iron concentrations maintained their normal limits. In contrast, serum iron concentrations increased by 0.43 ± 0.05 microg/dL in the patients receiving roxadustat, compared to the alpha erythropoietin group. Four patients (3 taking roxadustat and 1 receiving erythropoietin) received a rescue therapy (transfusion of a red blood cell concentrate, intravenous iron preparations and a stimulus of erythropoiesis). It should be emphasized here that 67 (32.8%) patients in the roxadustat group and 43 (43%) patients in the erythropoietin group also received oral iron preparations [18].

In addition, hepcidin levels were significantly reduced in the roxadustat group, compared to their baseline values (by 30.2 vs. by 2.3 ng/mL) in the alpha erythropoietin group [22] (See Table 2).

### 5.2. Lipids

In the study group of Chen et al. [16] the mean output level of total cholesterol concentration 390 was 172.8 ± 54.8 mg/dg/dL (4.5 ± 1.2 mmol/L) in the patients receiving roxadustat and 181.4 ± 49.0 mg/dg/dL (4.7 ± 1.3 mmol/L) in the placebo group (16). At week 9 of the therapy, a reduction in total cholesterol by 40.6 mg/dg/dL (1.0 mmol/L) was observed in the roxadustat group and by 7.7 mg/dg/dL (0.2 mmol/L) in the placebo group. In contrast, the mean output value of LDL cholesterol was 97.8 ± 34.0 mg/dL (2.5 ± 0.9 mmol/L) in the patients receiving roxadustat and 105.2 ± 42.2 mg/dL (2.7 ± 1.0 mmol/L) in the placebo group. After the 9th week of the study, the LDL cholesterol level was reduced by 25.3 mg/dL (0.7396 mmol/L) in the roxadustat group and by 5.8 mg/dg/dL (0.1 mmol/L) in the placebo group (28). Chen et al. found that, after 9 weeks of therapy, the roxadustat recipients in the group of patients non treated by dialysis had revealed a much greater decrease in lipid fractions (total and low-density lipoprotein cholesterol; *p* < 0.00001) [16].

In the patients with CKD, treated by dialysis in the study by Chen et al. [18], the output value of serum total cholesterol was 168.2 ± 42.9 mg/dg/dL (4.35 ± 1.1 mmol/L), LDL cholesterol was 95.1 ± 34.8 mg/dg/dL (2.45 ± 0.9 mmol/L), while in the alpha erythropoietin group, the output level of serum total cholesterol was 165.1 ± 41.4 mg/dg/dL (4.25 ± 1.05 mmol/L) and LDL cholesterol was 90.1 ± 29.4 mg/dg/dL (2.30 ± 0.75 mmol/L). In week 27, the study patients demonstrated a decrease in total cholesterol (difference in treatment vs. erythropoietin alpha) by −22 mg/dg/dL (−0.58 mmol/L) for total cholesterol and −18 mg/dg/dL (−0.47 mmol/L) for LDL cholesterol. A 14% improvement in the LDL:HDL (high density cholesterol) ratio was achieved in the patients from the roxadustat group, compared to those receiving alpha erythropoietin. The difference in serum triglycerides in the roxadustat group, compared to the erythropoietin alpha group, was −12.4 ± 9.7 mg/dg/dL (−0.14 ± 0.11 mmol/L) [18] (See Table 2).

### 5.3. C-Reactive Protein (CRP)

The parameters of inflammation were determined by the C-reactive protein (CRP). In the roxadustat group, 46 out of 204 patients had elevated CRP levels, above the upper limit of the normal range. In the erythropoietin alpha group, 20 out of 100 patients demonstrated CRP levels, elevated above the upper limit of the normal range. In subsequent concentration assays, no significant differences in CRP variations were observed between the patients with normal baseline CRP concentrations and those with elevated baseline CRP levels [18]. However, the patients in the alpha erythropoietin group with elevated CRP levels had a worse response to the drug and a lower increase in hemoglobin levels. That observation confirms the thesis that inflammation affects erythropoiesis. In turn, the increased concentration of inflammation parameters did not affect the increase in hemoglobin in the patients treated with roxadustat, which was suggested after the second phase trials of the drug.

## 6. Summary

The presented studies show that roxadustat, when administered in patients with terminal renal failure, significantly increases the level of hemoglobin, affects iron metabolism, increases iron absorption from the gastrointestinal tract and reduces the level of cholesterol, while not increasing blood pressure.

Phase III studies of roxadustat treatment were conducted in patients with CKD not treated or treated with renal replacement therapy (hemodialysis, peritoneal dialysis).

The studies, which compared the effects of roxadustat to those of placebo [22,23,24,25,26,29] demonstrated a significant increase in hemoglobin concentrations after roxadustat, both in conservatively treated and dialyzed patients.

The studies which juxtaposed the effects of roxadustat with those of erythropoietin [22,25,27,29,30] or darbapoietin [28], showed that, regarding changes in hemoglobin levels, the effects of roxadustat were not worse, while being usually better than the effects of erythropoietin

### 6.1. Adverse Events after Roxadustat

It has been shown that in Phase 2 trials involving CKD patients treated with dialysis who were treated with roxadustat (administered three times a week), the tolerance profile was similar to that of patients with CKD receiving dialysis and erythropoietin alfa [9]. Treatment-related serious adverse events (SAEs) were reported in 24% of patients treated with roxadustat and 17% of patients in the placebo group. The authors also analyzed the risk of a composite cardiovascular safety point (including death, myocardial infarction, stroke, hospitalization, heart failure, unstable angina requiring hospitalization, or thromboembolism) in patients after 19 weeks of treatment. The events described above occurred in 12% of patients treated with roxadustat compared with 17% of patients treated with erythropoietin alfa. There were three deaths in the group treated with roxadustat, none of which were related to the treatment [8].

Another phase 2 study (NCT01596855) [33], in which patients with CKD were studied, showed similar results. Treatment-related adverse events were reported in 43% (32/74) of roxadustatat-treated patients and 18% (4/22) of erythropoietin alfa-treated patients. The most common side effects (occurring in >5% of the subjects) were decreased appetite and muscle cramps (7% vs. 5% and 5% vs. 14% respectively, after treatment with roxadustat and erythropoietin alfa). In the Phase 2b trial in patients undergoing dialysis treatment (NCT01414075), roxadustat was a well-tolerated drug (24): back pain (5% vs. 4%), fatigue (5% vs. 0%) and hyperkalemia (5% vs. 0%).

In phase III trials, roxadustat was a well-tolerated drug in both dialyzed [18,20] and non-dialyzed [16,32] patients with CKD. In these studies, the most common treatment-related adverse events (AEs) after roxadustat were nasopharyngitis, back pain, diarrhea and vomiting [20,32]. We would like to emphasize here that hyperkalemia and infections of the upper respiratory tract occurred more often in the group of patients treated with roxadustat, and hypertension was more common in the group of patients treated with epoetin alfa [22].

In patients with CKD, the tolerance profile of roxadustat was similar to that of darberythropoietin [32]. Overall, the summary information on the safety of the treatment used, be it roxadustat or erythropoietin alfa, to the information on serious cardiovascular (CV) adverse events was similar. In patients treated with roxadustat, the most common adverse events were diarrhea, hypertension, pneumonia, headache and arteriovenous fistula thrombosis. Additional serious adverse events include sepsis and acute myocardial infarction.

Among the SAEs that occurred, in four patients treated with roxadustat, there were disturbances in vascular access to dialysis, hip fractures, non-cardiac pain in the chest and dyspnoea, and in one patient in the placebo group. Overall, treatment-related SAEs occurred in 13% (8/61) of roxadustat-treated patients and in 13% (4/30) of 471 placebo-treated subjects. We would like to emphasize that neither of them was found to be related to treatment [33]. A study of patients with CKD (NCT01244763) confirmed a good tolerance profile of roxadustat, and no treatment-related SAEs were reported [11].

### 6.2. Potential Benefits of Roxadustat, Assumed after Clinical Trials with CKD Patients, Both Non-Dialyzed and Dialyzed

The advantages of roxadustat include: (1) the increase of hemoglobin levels, (2)the maintenance of elevated Hb levels, (3) increased endogenous EPO expression within the physiological range, (4) positive effects on iron metabolism, e.g., by reducing the blood levels of hepcidin, (5) increased iron absorption in the gastrointestinal tract, (6) no inflammation-inducing effects in the body, (7) easy oral administration,(8) reversible and transient inhibition of HIF-PHD, (9) not increased blood pressure, (10) lowered cholesterol and(11) neither high levels of EPO nor side effects of iron supplementation.

### 6.3. Roxadustat Effects in Patients with Diabetes and Obesity

Following a thorough review of the results provided by the previously presented randomized trials in phase 2 and 3 [33,34,35,36,37,38], a question arises whether the effect of roxadustat (hemoglobin level and the number of red blood cells) is the same in patients with or without diabetes and with or without obesity. The authors of the above-mentioned studies did not take into account the separate effects of roxadustat on the level of hemoglobin and the number of erythrocytes in patients with or without diabetes and with or without obesity. After carefully reading the descriptions of particular study groups, it appears that the average study group may have included approximately 24–42% of patients suffering from diabetes. Many of the examined patients also suffered from obesity.

Analyzing the most recent data, it appears that roxadustat may have a beneficial pleiotropic effect, regardless of its primary effects, in patients with diabetes and obesity.

In obese people, blood flow through the organs is reduced and oxygen consumption is increased. These phenomena lead to insufficient tissue oxygenation. Insufficient tissue oxygenation leads to secretion of HIFs. Increased secretion of HIF-1α leads to the development of insulin resistance and other metabolic disorders. Increased concentration of HIF-1α is accompanied by intensification of inflammatory processes and fibrosis in obese subjects. Contrary to what we wrote, HIF-2α plays a protective role against the development of diabetes (of which HIF-1α is involved in the pathogenesis). Well-conducted pharmacological modulation of HIF activity may contribute to the effective fight against obesity and diabetes.

In order to find answers to the questions about the effectiveness of roxadustat in diabetic and obese patients, we need time and further studies.

### 6.4. New Clinical Trials of Roxadustat in Patients with CKD

Phase 3 clinical trials are currently underway on the efficacy and safety of roxadustat. These include:

1. DOLOMITES study (NCT02021318). It is a randomized, open-label, Phase 3 study that compares the anemia control efficacy and safety of roxadustat versus darberythropoietin in 616 patients with non-dialyzed CKD.
2. In a randomized, open-label, phase 3 trial by Japanese authors (NCT02988973), the observation of CKD patients was not treated with renal replacement therapy, comparing the efficacy and safety of roxadustat versus darberythropoietin. Subjects were previously treated with recombinant human EPO or darberythropoietin alfa. Another study to be enrolled in 325 will evaluate the efficacy of anemia correction and the safety of roxadustat in patients who have previously been treated with beta erythropoietin. The primary endpoint of this study will be the ocean of change in Hb levels after 18–24 weeks of treatment.
3. An open-label, phase 2/3 extension study (NCT01630889) assesses the long-term efficacy of anemia control and the safety of roxadustat in patients with CKD not treated and treated with dialysis.
4. Preparations are underway to start a randomized, double-blind, placebo-controlled, phase III study (NCT03263091). The aim of the research is to assess the efficacy and safety of roxadustat in the treatment of anemia in patients at low risk of myelodysplastic syndrome MDS and not receiving frequent blood transfusions. The authors plan to include 184 patients with CKD in the study. The authors set the main goal of the study as not to transfuse blood for ≥56 days.
5. Currently, 175 patients with CKD are enrolled in the Phase 2/3 study (NCT03303066), which aims to assess the efficacy (percentage of people with an Hb increase that allows life without transfusion) and the safety of roxadustat in a lower risk of MDS. The study is to be completed by the end of 2020.

## 7. Conclusions

The two main causes of developing renal anemia in patients with CKD are significantly reduced EPO synthesis and significant disturbances in iron metabolism. The recognition and treatment of renal anemia among physicians in clinical practice is still low and inadequate [39]. Roxadustat, as the first drug from the HIF-PHI group, causes an increase in the concentration of endogenous EPO synthesis and a significant reduction in hepcidin concentration. The conducted studies also showed that roxadustat also raises Hb levels in patients with an ongoing inflammatory process (no similar effect was found in ESA patients). Taking this into account, it should be stated that roxadustat gives the possibility of a new approaches to treating anemia in patients with CKD. Collected data suggest that roxadustat is an effective drug in anemia in patients with CKD. More long-term clinical trials are needed to better assess the efficacy and safety of roxadustat, its application and its pleiotropic effects.

## Figures and Tables

**Table 1:** Summary of the results of phase 2 studies of roxadustat use in patients with renal failure not undergoing dialysis [NDD].

NCT	Patients	Basal Hb (g/dg/dL)	S/R/C	Effect—Change Hemoglobin Level (g/dg/dL)	Adverse Events (%)	Serious Adverse Events (%)	Reference
01599507	NDD/Placebo	<10.0	156/91/82	After 8 weeks: Low dose roxa—1.82+/−0.21 High dose roxa—2.59+/−0.26 Placebo—0.65+/−0.13	Roxa—59 Placebo—63	Roxa—13.1 Placebo—13.3	[9]
00761657	NDD/Placebo	NDD treated roxa:	293/88/28	After 6 weeks therapy:	Roxa—59.1 Placebo—59.1	Roxa—5 Placebo—4	[13]
		0.7 mg/kg—10.3		0.7 mg/kg—+0.4			
		1.0 mg/kg—10.4		1.0 mg/kg—+0.4			
		1.5 mg/kg—10.3		1.5 mg/kg—+1.2			
		2.0 mg/kg—10.3		2.0 mg/kg—+1.8			
		Placebo—10.3		Placebo—0.1			
01244763	NDD	<10.5	357/145	After 16(A and B) and after 24 weeks roxa therapy: A (1 mg/kg)—1.71+/−0.21 B (1.7 mh/kg)—1.09+/−0.21 C 50 mg—0.57+/−0.21 D 100 mg—1.53+/−0.20 E 70/100/150—0.77+/−0.20 F 70 mg—0.61+/−0.20	80.0	24.1	[11]
01964196	NDD/Placebo	<10.0	190/80/27	After 6 weeks. Rate of rise (g/dg/dL/week) compare to placebo	Placebo 70.4	Placebo 7.4	[21]
				Roxa 50 mg/TIW—+0.254	Roxa 50 mg/TIW—74.1	Roxa 50 mg/TI—22.2	
				Roxa 70 g/TIW—+0.508	Roxa 70 mg/TIW—88.5	Roxa 70 mg/TIW—0	
				Roxa 100 g/TIW—+0.623	Roxa 100 mg/TIW—74.1	Roxa 100 mg/TIW—74.1	

S/R/C—Screened/Randomized/Control, NDD/Placebo—patients not treated by hemodialysis/placebo, roxa—roxadustat, TIW—three times a week.

**Table 2:** Additional assessed parameters–hepcidin, total and LDL-cholesterol.

	Hepcidin		Total Cholesterol		LDL—Cholesterol		
	Roxadustat	EPO	Roxadustat	EPO	Roxadustat	EPO	Reference
Output	95.89	114	172.8	181.4	97.8	105.2	[16]
Change	−56.14	−15.10	−40.7	−7.7	−25.3	−5.8	
Output	180	148	168	165	95.1	90.1	[18]
Change	−30.3	−2.2					

## Data Availability

Data sharing not applicable.
